# Investigating the Crucial Aspects of Developing a Healthy Dormitory based on Maslow’s Hierarchy of Needs—A Case Study of Shenzhen

**DOI:** 10.3390/ijerph17051565

**Published:** 2020-02-28

**Authors:** Zezhou Wu, Lei Liu, Shenghan Li, Hao Wang

**Affiliations:** 1 Sino-Australia Joint Research Centre in BIM and Smart Construction, Shenzhen University, Shenzhen 518060, China; wuzezhou@szu.edu.cn; 2 Department of Construction Management and Real Estate, College of Civil and Transportation Engineering, Shenzhen University, Shenzhen 518060, China; leiliu940610@163.com; 3 School of Management Science and Engineering, Central University of Finance and Economics, Beijing 100081, China; holy.wong@connect.polyu.hk

**Keywords:** healthy dormitory, crucial aspect, Maslow’s hierarchy of needs, measurement indicator, structural equation modeling

## Abstract

In recent years, with the development of green building and the increase of health awareness, the concept of healthy building has been proposed. Recently, studies have been made on developing healthy residential buildings; however, few attentions have been paid to the development of healthy dormitories. To bridge this research gap, this paper aims to investigate the crucial aspects of developing a healthy dormitory. Based on the Maslow’s hierarchy of needs, three influencing aspects which include 17 measurement indicators are identified. Questionnaire surveys are subsequently conducted to collect students’ perceptions on the identified indicators. After a structural equation modeling (SEM) analysis, the relationships between the three influencing aspects are analyzed. The research findings show that building performance, bodily sensation, and humanistic environment must be taken into account in the development of a healthy dormitory. In addition, it is revealed that building performance has a significant impact on bodily sensation, while bodily sensation has a significant impact on humanistic environment. However, building performance is found having little impact on humanistic environment. The findings of this study could provide useful information for the construction of healthy dormitories.

## 1. Introduction

In recent years, with the rapid economic development in China, sustainability and health problems have been attracting attentions from both the central government and the public [1,2,3]. As human beings spend nearly 90% of time indoors, the indoor environment could significantly influence the human beings’ health status [4]. In this circumstance, the buildings which can provide healthy, comfortable and safe living environment for human beings are highly required [5]. To facilitate the development of such buildings, a new concept of “healthy building” has been proposed by the Architectural Society of China (ASC) [6].

According to the “Assessment Standard for Healthy Building” published by the ASC, “healthy building” is defined as the buildings that not only fulfill the basic functional requirements but also have the abilities of providing healthier environment, facilities and services to the users to protect their physical and psychological health [6]. A healthy building takes the concept of green building as a premise, while the focus turns from the building itself to the residents of the building. Mao, et al. [7] claimed that a health building should be based on not only the physical aspects, but also the psychological factors. According to the given definition, the purpose of healthy buildings is very similar with WELL (the world’s first building standard focusing exclusively on human health and wellness) labeled buildings, namely providing a more comfortable environment for people to protect their health, work efficiency, concentration, etc. [8].

In the last years, several models have been proposed to assess a “healthy” indoor environment, in particular on the indoor environmental quality (IEQ) [9]. Piasecki [10] implemented the IEQ model which includes elements of thermal comfort, indoor air quality, acoustic comfort and daylight quality to evaluate a single-family building. Meanwhile, specific aspects of the indoor environment have been investigated. For example, Piasecki and Kostyrko [11] developed a model to assess the indoor air quality (IAQ) as they regarded that IAQ is one of the most important aspects affecting a building user’s comfort and satisfaction. Based on identifying the evolution of the WELL Building Standard, Alfonsin, et al. [12] introduced the active strategies for designing a healthy building. McLeod [13] explored the intersection of physical activity and the built environment based on the WELL Building Standard.

From the literature review, the current studies mainly focused on office buildings or residential buildings, studies on dormitory buildings are very few. According to the statistics provided by Fan [14], the area of dormitory buildings accounted for 35% of the total building area of a campus. Besides, it is estimated that the college students spend 80.4% of their time indoors, while 50.4% in dormitories [15]. Moreover, it is predicted that the energy consumption of dormitory accounted for 18% of the total energy consumption in college, and the per capita energy consumption of students is much higher than that of the national average level [16]. According to the findings revealed by Petidis, et al. [17], campus dormitories have many common problems, such as poor indoor thermal environment, insufficient lighting, high humidity, insufficient ventilation and promoting poor interpersonal relationships, which has a negative effect on students’ life. Owning to the demonstrative and educational functions of buildings in campus [18], it is of great necessity to develop healthy dormitories to improve the living environment of students.

To improve the living environment of campus residents, efforts have been extensively made. In 2013, Kilicaslan [19] identified five concerned points about dormitory construction, such as physical conditions of dormitory rooms, study environments in dormitories, functionality of wet areas, socialization, and suggestions. Ding, et al. [20] emphasized the importance of behavioral changes to dormitory energy conservation, and developed an agent-based model to simulate energy-saving scenarios under different strategies. Additionally, He, et al. [21] investigated the thermal comfort of air-conditioned dormitories in hot and humid climate areas. The results showed that students have a strong dependence on air conditioning, and they have psychologically improved the ability to adapt to the high temperature and humidity. Lei, et al. [22] analyzed the influence of natural ventilation on the thermal comfort of dormitory through CFD simulation, and provide some guidance for the improvement of indoor environment. In order to get a better understanding of college students’ exposure to PM2.5 and the associated health risk, Wang, et al. [23] measured the PM2.5 mass concentrations in dormitories and outdoor environments in a university in Nanjing, China, in 2016–2017. In addition, Frijters, et al. [24] studied the influence of random allocation of dormitory on students’ physiology and psychology, and it turns out that the peer effect is very strong. Pilechi and Taherkhani [25] suggested renovating the traditional public baths in Iranian university dormitories to serve as a cultural gathering place for social activities, to help strengthen the communication among students, solve social problems and improve their life quality.

From the above literature review, it can be seen that achievements have been made in the development of healthy dormitory; however, there is no consensus on the importance of influencing factors in developing a healthy dormitory. To bridge this research gap, this study adopts the Maslow’s hierarchy of needs to identify the crucial influencing aspects.

## 2. Theoretical Background

This section introduces the theoretical background of this study. The explanations of Maslow’s hierarchy of needs (MHN) are firstly presented. This is followed by the description of the proposed theoretical model.

### 2.1. Maslow’s Hierarchy of Needs

**Figure 1:**
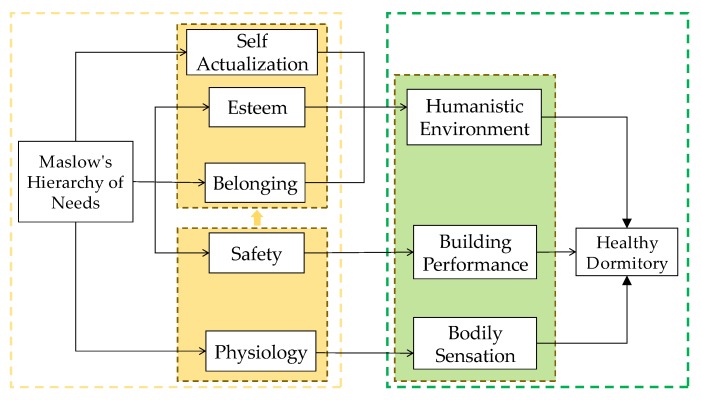
Definition framework of healthy dormitory.

Maslow’s hierarchy of needs divides human needs into five categories from low to high, i.e., physiological needs, safety needs, belonging, esteem and self-actualization [26]. The physiology needs (including air, water, food, shelter, sleep, clothing, reproduction) and the safety needs (including personal security, employment, resources, health, property) are categorized as low-level demands, while the “belonging” (including friendship, intimacy, family, sense of connection), the “esteem” (including respect, self-esteem, status, recognition, strength, freedom) and the “self-actualization” (including realizing personal potential, self-fulfillment, seeking personal growth and peak experiences) are regarded as higher levels [27]. It is assumed that only when the lower level needs are met can they be motivated to pursue the higher level needs [28]. Having healthy dormitories, aiming at the health needs of college students for their residential environment, is consistent with the Maslow’s hierarchy of needs. Based on the classical Maslow’s hierarchy of needs, a definition framework of healthy dormitory is constructed, consisting of three potential influencing aspects, i.e., bodily sensation (the health level of students’ physiological system, including air, comfort and nutrition), building performance (the green and healthy effects of architecture, such as ventilation, daylighting and energy-saving), and humanistic environment (the interactive connection between students, spiritual needs, and macro public policies in dormitory area), as shown in Figure 1.

To measure the potential influencing aspects, measurement indicators were selected through a comprehensive literature review. A total of 17 measurement indicators were obtained, as shown in Table 1.

### 2.2. Theoretical Model

**Figure 2:**
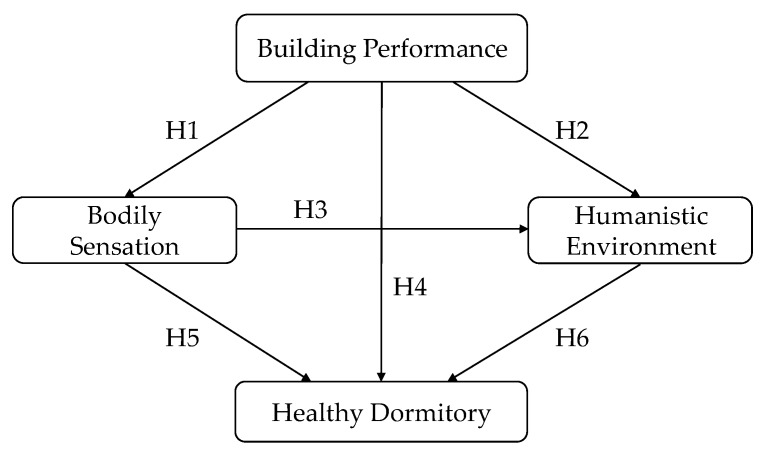
Preliminary theoretical model.

Based on the literature review, certain relationships between the potential influencing aspects can be assumed. For example, Cuce, et al. [57] concluded that single-side ventilation and cross-ventilation can have good effect on cooling and improving air quality in school buildings, with different functions as long as the height and depth of rooms are properly designed. Kisilewicz, et al. [58] found that effective thermal insulation of the external partitions could supply consistent shading of the windows against direct solar radiation, providing thermal comfort to the users. Thus, it is assumed that “Building Performance” has a significant positive impact on “Bodily Sensation”. A preliminary theoretical model was developed, as shown in Figure 2. The following hypotheses are proposed: H1: “Building Performance” has a significant positive impact on “Bodily Sensation”; H2: “Building Performance” has a significant positive impact on “Humanistic Environment”; H3: “Bodily Sensation” has a significant positive impact on “Humanistic Environment”; H4: “Building Performance” has a significant positive impact on the development of health in the dormitory; H5: “Bodily Sensation” has a significant positive impact on the development of health in the dormitory; H6: “Humanistic Environment" has a significant positive impact on the development of health in the dormitory.

## 3. Research Methodology

This section introduces the research methodology used in this study. The process of data collection is firstly presented. Then, the description of statistical analysis is provided.

### 3.1. Data Collection

In order to test the proposed research hypotheses, a questionnaire survey was implemented. An initial questionnaire was designed and submitted to three experts for review. Then, 20 students were invited to conduct a pilot study to eliminate any ambiguity and incomprehensibility. Finally, the formal questionnaire was determined, as shown in the Supplemental Materials. The first part collects the basic information of the respondents. The second part deals with the measurement of the three constructs, and the proposed constructs were measured by items evaluated on 5-point Likert scales, where ‘‘1” = strongly disagree, ‘‘2” = disagree, ‘‘3” = neutral, ‘‘4” = agree, and ‘‘5” = strongly agree.

The buildings investigated in this study are the existing dormitories in Shenzhen University, such as Liyuan (built in 1980s), Xiyuan (built in 1990s), Qiaoyuan (built in 2000s) and Nanyuan (built in 2010s). The basic structure of the four buildings are reinforced concrete, and there are no mechanical ventilation facilities indoor. The questionnaires were distributed during 29 July 2019 and 6 August 2019. A total of 375 questionnaires were collected, of which 344 responses were valid.

### 3.2. Data Analysis

The data analysis process includes two parts. First, the Statistical Product and Service Solutions (SPSS) was used to analyze the quality of questionnaire data [59], such as reliability and validity. Then, a structural equation modeling analysis is carried out by using the software of AMOS 22.0 [60].

#### 3.2.1. Reliability and Validity Analysis

Internal consistency reliability was used to measure the accuracy, stability and consistency of the questionnaire. Cronbach’s α is a crucial index. Generally, α > 0.8 indicates excellent internal consistency [61,62]. The corrected item-total correlations (CITCs) are used to test the reliability of scale. Li [63] specified that CITCs should be greater than 0.35. In this study, the CITCs standard is defined as greater than 0.4. In addition to reliability analysis, validity analysis is one of the important scale accuracy tests as well, referring to the measurement tool can accurately evaluate the degree of indicators’ characteristics [64]. In this study, structural validity, convergent validity and discriminant validity were adopted. Since the three potential dimensions of healthy dormitory had been determined based on a large review of literature, confirmatory factor analysis (CFA) was used for structural validity. The test value standard of Kaiser–Meyer–Olkin (KMO > 0.5) and Bartlett tests (P ≤0.05) must be met firstly. Then, a cross validation was carried out through the confirmatory factor analysis. The fit indices of χ2/df, goodness of fit index (GFI), adjusted goodness of fit index (AGFI), comparative fit index (CFI), parsimony goodness of fit index (PGFI), normed fit index (NFI), root mean square error of approximation (RMSEA), and standardized root mean square residual (RMR) were used to confirm the measurement models [65]. The composite reliability (CR) and the average variance extraction (AVE) were used to test the convergent validity of the questionnaire. CR > 0.6 and AVE > 0.5 indicate the combination validity of the model is good.

#### 3.2.2. Structural Equation Modeling

After reliability and validity analysis, the structural equation modeling (SEM) was employed. The SEM can be used to establish, estimate and test the relationships between variables [66]. A structural equation model usually consists of two sub-models: (1) measurement model, which describes the relationship between potential variables and observed variables; (2) structural model, which defines the relationship pattern between unobservable factors (endogenous and exogenous variables) [67]. In this study, maximum likelihood (ML) was used to estimate the parameters of structural equation models. Assessments of model fitting were performed based on inferential goodness of fit indices and other descriptive and alternative indices.

**Figure 3:**
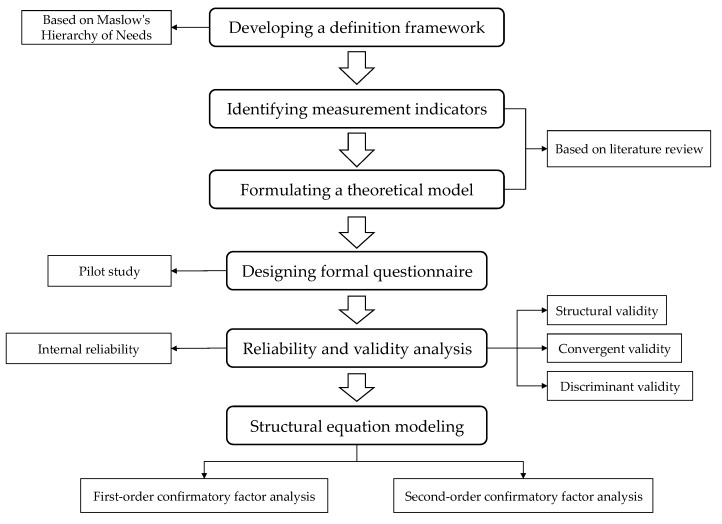
Research process of this study.

The research process of this study is shown in Figure 3.

## 4. Results and Discussions

This section firstly presents the results of statistical analysis. Discussions are further made based on the derived results.

### 4.1. Reliability and Validity Results

**Figure 4:**
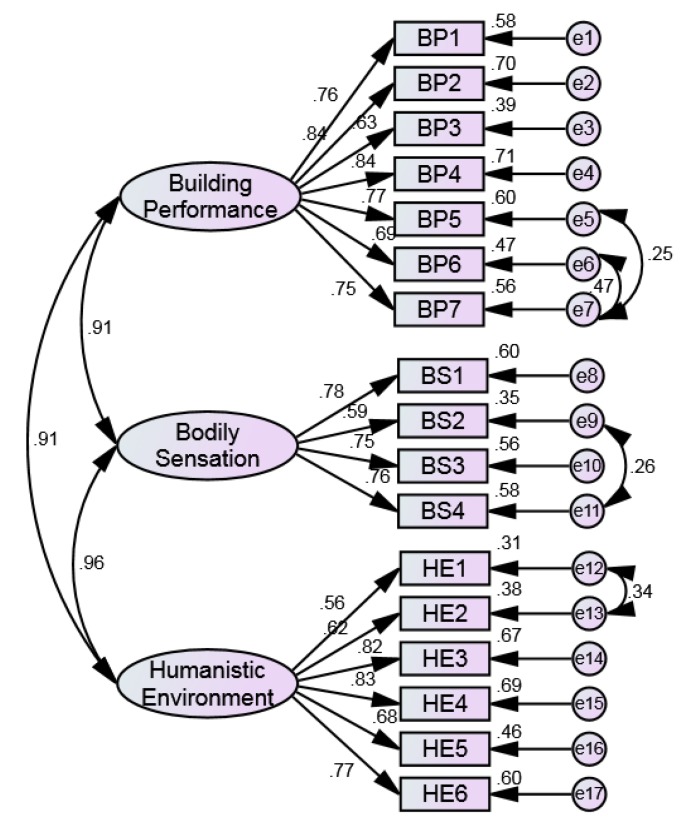
Standardized regression weights of the measurement model.

The reliability and validity results were derived by SPSS and AMOS, as shown in Table 2. The Cronbach’s α of each dimension was greater than 0.8, and the total scale was 0.917. The CITCs of all items were higher than 0.4, which indicates that there is a good correlation between items. Therefore, the reliability of the questionnaire met the acceptable standard. In addition, the KMO value was 0.932, and the *p*-value of the Bartlett test was 0.000, which shows a strong correlation among variables. According to the factor analysis results obtained by principal component extraction method, 0.5 was selected as the critical value of factor loading. Because the factor loads and the variance of common factors were all greater than 0.5, 17 factors were retained. Confirmatory factor analysis (CFA) was further conducted. The measurement model of CFA was established, as shown in Figure 4. The results of parameter estimation showed that the model is in good agreement with data. Finally, the CR of latent variables were greater than 0.6, the AVE were greater than 0.5, and the square root of the mean variance extraction quantity of the latent variables was larger than the correlation coefficient of the variable and other variables. The results showed that the questionnaire has good convergent validity and discriminant validity, as shown in Table 3.

### 4.2. First-Order Confirmatory Factor Analysis

**Figure 5:**
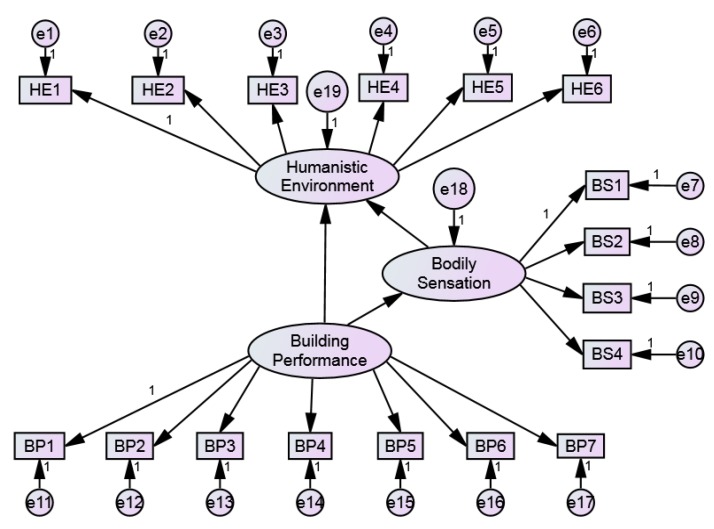
Initial model.

The preliminary structural equation model for first-order confirmatory factor analysis was established to test the three hypotheses proposed, i.e., H1: “Building Performance” has a significant positive impact on “Bodily Sensation”; H2: “Building Performance” has a significant positive impact on “Humanistic Environment”; H3: “Bodily Sensation” has a significant positive impact on “Humanistic Environment”, as shown in Figure 5. The results showed that some fitting indexes of the initial model are not up to standard, and it needs to be modified appropriately. Model modifications include Model Building and Model Trimming [67]. When the path coefficient (P) is greater than 0.05, it indicates that the path has a negative affect and should be deleted.

**Figure 6:**
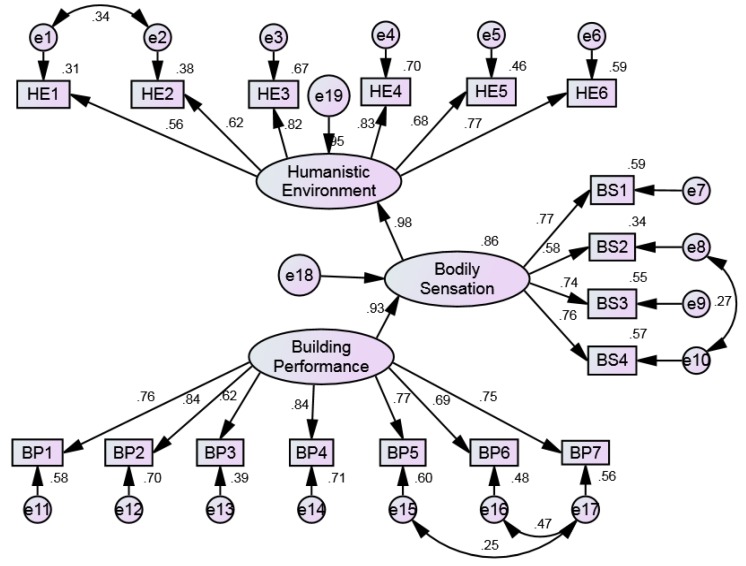
Standardized estimation of the first-order confirmatory factor analysis.

According to the regression weights in the initial model, the hypothetical path of “Humanistic Environment ← Building Performance” with *p*-value greater than 0.05, should be deleted. Furthermore, the initial model will be further extended by modification index. Double arrow (↔) means that adding a correlation path between two variables reduces the value of χ^2^/df at least. There are high correction indices between HE1 and HE2, BS2 and BS4, BP5 and BP7, and BP6 and BP7. Thus, these paths should be added. Finally, the standardized estimation of the first-order confirmatory factor analysis which meets the requirements of the evaluation criteria is obtained, as shown in Figure 6. The model evaluation parameters are shown in Table 4.

### 4.3. Second-Order Confirmatory Factor Analysis

**Figure 7:**
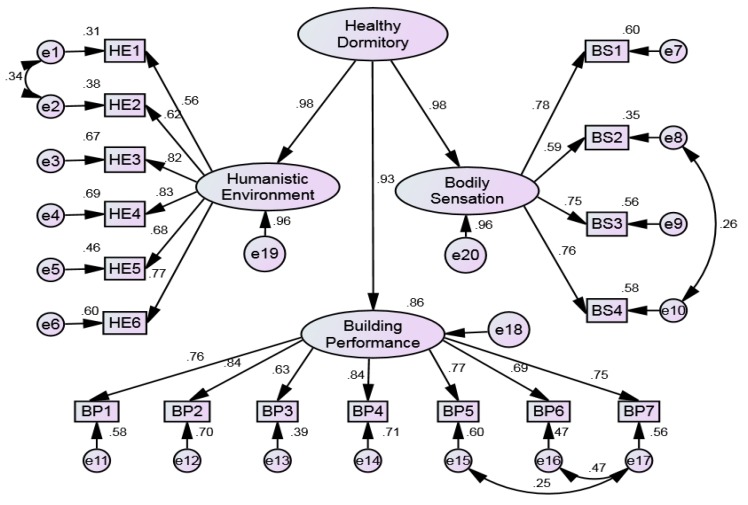
Standardized estimation of the second-order confirmatory factor analysis.

Since healthy dormitory is composed of three latent variables, and it is necessary to establish an improved structural equation model. The structural equation model for second-order confirmatory factor analysis was established to test the other three hypotheses, i.e., H4: “Building Performance” has a significant positive impact on the development of health dormitory; H5: “Bodily Sensation” has a significant positive impact on the development of health dormitory; H6: “Humanistic Environment” has a significant positive impact on the development of health dormitory. The improved model is shown in Figure 7, and the statistical results are shown in Table 5. It can be found that the CR values of all latent variables were greater than 1.96, and *p*-values were lower than 0.005. Therefore, three potential variables had significant positive impacts on the healthy dormitory development.

Based on the statistical data of first-order and second-order confirmatory factor analysis, the verification results of all hypothesis are shown in Table 6.

### 4.4. Discussions

The verified results showed that the three potential influencing aspects have significant positive effects on healthy dormitory development, and they are also internally related. The results are further discussed by emphasizing the relationships between the three influencing aspects and the influences of the three crucial aspects in healthy dormitory development.

#### 4.4.1. Relationships among Building Performance, Bodily Sensation and Humanistic Environment

From the results of the six proposed hypotheses, it was surprising that H2 is not supported, which assumed that the building performance has a significant positive impact on the humanistic environment. This assumption was proposed based on the previous literature. For example, based on an assessment that integrated historical research across disciplines, Hoisington, et al. [68] offered 10 questions that highlight the importance of current lessons learned regarding the architectural environment and humanistic policies. Evans [69] reviewed the literature on the relationship between the building and mental health (characterized by psychiatric disorder, symptoms of psychological distress, and difficulties with self-regulation) and further discussed the effects of the building on stress, behavioral control, and levels of social support. These two studies both showed that architecture structure affects the humanistic environment. However, according to the results revealed in this study, it was found that there is no positive relationship between building performance and humanistic environment. The underlying reason may be, although they both aim to improve students’ mental health, the perspectives are different. Building performance improves physical comfort firstly and thereby indirectly enhance mental health, while the humanistic environment improves mental health from interpersonal connections, such as group activities, psychological counseling and policy implementation. An in-depth interview with 15 students and 5 experts confirmed the speculation. They emphasized that dormitory is only a place providing interactive activities, while the humanistic environment mainly focuses on the interactive feelings among students. In addition, it is found that bodily sensation serves as an intermediary between building performance and humanistic environment, that is, building structure affects bodily sensation, and thereby partly affects humanistic environment. This causality is in line with the development of healthy buildings currently.

#### 4.4.2. Influences of Three Crucial Influencing Aspects in Healthy Dormitory Development

Results showed that building performance, bodily sensation and humanistic environment had significant positive effects on developing a healthy dormitory, and the standardized path coefficients were 0.93, 0.98 and 0.98, respectively. It is very curious why the three aspects have such a high impact on dormitory compared with the office and residential buildings. By visiting the dormitory, it can be understood that there are usually four to eight students living in a dormitory, and the area of the dormitory is about 24 m^2^, indicating the per capita area is between 3 m^2^ and 6 m^2^. Compared with other types of buildings, such an area is so narrow that is easy to affect students’ physical health. Based on the safety and thermal comfort of students, it is inappropriate for dormitory implemented in accordance with the construction requirements of the residential healthy buildings. The requirements of building performance and thermal environment should be improved. Therefore, the building performance and the bodily sensation are more important for the development of healthy dormitory than other buildings. In addition, compared with other building types, the humanistic environment is also important for the development of healthy dormitory. The difficulties in interpersonal relationships, academic pressure, social adaptation, employment prospect and other aspects make college students susceptible to various psychological and behavioral problems, and the severity has an increasing trend [70]. Therefore, these three influencing aspects are crucial in developing healthy dormitories.

## 5. Conclusions

Dormitory building, as an important and special building type, has not been emphasized in the development of healthy buildings. Therefore, this study aimed to investigate the crucial aspects of healthy dormitory development. Based on the Maslow’s hierarchy of needs, three potential influencing aspects, such as building performance, bodily sensation, and humanistic environment, were identified. Then, the relationships among the three identified aspects and their effects on healthy dormitory development were investigated by using structural equation modeling. Results showed that building performance has a positive impact on bodily sensation, and bodily sensation has a positive impact on humanistic environment. In addition, it is found that all of the three identified aspects have positive impacts on heathy dormitory development.

Student-oriented planning is the core of developing a healthy dormitory. This study is the first attempt that introduces a classical demand behavior theory in healthy dormitory development. The research method implemented in this study can also be employed in other regions or countries to investigate the local crucial aspects of healthy dormitory development. However, as this is the first time that the Maslow’s hierarchy of needs is introduced in healthy dormitory development, there is no mature measurement scales in the existing literature. Future research is suggested to be carried out in a wider range of cities with further developed measurement scales.

## Figures and Tables

**Table 1:** Measurement indicators.

Influencing Aspects	Measurement Indicators	References
Building Performance	Acoustic environment (BP1)	Pinho, et al. [29] ISO 1996 [30]
	Gas tightness (BP2)	Alfano, et al. [31] ISO 9972 [32]
	Shading effect (BP3)	Charde and Gupta [33] Boubekri and Lee [34]
	Natural ventilation (BP4)	Asfour [35]
	Light utilization (BP5)	Bellia, et al. [36] ISO 10,916 [37]
	Intelligent energy consumption monitoring system (BP6)	Yoon, et al. [38] Wei, et al. [39] ISO 23,045 [40]
	Life-cycle cost (BP7)	Arpke and Strong [41] Huang, et al. [42]
Bodily Sensation	Thermal comfort (BS1)	Aguilera, et al. [43] Alfano, et al. [44] ISO 1055 [45]
	Biophilia (BS2)	Kellert [46] Xue, et al. [47]
	Feeling of air quality (BS3)	Andersen, et al. [48] Wei, et al. [49] Radwan and Issa [50]
	Nutrition (BS4)	Krešić, et al. [51]
Humanistic Environment	Daily-life convenience (HE1)	Lu, Ge, Chen, Qu and Chen [18]
	Regional collective characteristic (HE2)	Pilechi and Taherkhani [25]
	Dormitory optional mechanism (HE3)	Frijters, Islam and Pakrashi [24] Shook and Clay [52]
	Education and publicity (HE4)	Baihua [53] Zhao and Zhu [54]
	Policies diversification (HE5)	Jian [55]
	Innovative progress (HE6)	Pedaste, et al. [56]

**Table 2:** Reliability and validity analysis.

Factor	Item (Factor Loading)	CITC	Cronbach’s α	KMO and Bartlett’s Test	Model Fit
Building Performance	BP1(0.76)	0.723	0.905	KMO = 0.940 Approx. Chi-Square = 2406.707 Df = 136 *p* = 0.000	χ^2^/df = 2.072 RMSEA = 0.072 RMR = 0.029 GFI = 0.885 AGFI = 0.840 CFI = 0.950 PGFI = 0.636 NFI = 0.908
	BP2(0.84)	0.780			
	BP3(0.63)	0.617			
	BP4(0.84)	0.778			
	BP5(0.77)	0.722			
	BP6(0.69)	0.668			
	BP7(0.75)	0.713			
Bodily Sensation	BS1(0.78)	0.744	0.824		
	BS2(0.59)	0.580			
	BS3(0.75)	0.699			
	BS4(0.76)	0.737			
Humanistic Environment	HE1(0.56)	0.568	0.860		
	HE2(0.62)	0.593			
	HE3(0.82)	0.758			
	HE4(0.83)	0.767			
	HE5(0.68)	0.635			
	HE6(0.77)	0.753			

**Table 3:** Parameters of convergent and discriminant validity.

	CR	AVE	Building Performance	Bodily Sensation	Humanistic Environment
Building Performance	0.904	0.575	0.758		
Bodily Sensation	0.809	0.517	0.306	0.719	
Humanistic Environment	0.868	0.529	0.231	0.225	0.727

**Table 4:** Comparison of fitting degree between initial hypothesis model and modified model.

Fit Index	χ^2^/df	RMR	RMSEA	GFI	AGFI	CFI	PGFI	NFI
**Ideal value**	<3	<0.05	≤0.08	>0.8	>0.8	>0.9	>0.5	>0.9
**Initial test data**	3.142	0.035	0.102	0.824	0.768	0.893	0.625	0.853
**Corrected test data**	2.062	0.029	0.071	0.885	0.841	0.950	0.642	0.908

**Table 5:** Regression weights of the second-order confirmatory factor analysis.

			Estimate	S.E.	C.R.	*p*	Label
Humanistic Environment	←	Healthy Dormitory	0.734	0.098	7.523	***	par_15
Building Performance	←	Healthy Dormitory	1				
Bodily Sensation	←	Healthy Dormitory	0.93	0.087	10.713	***	par_16
HE5	←	Humanistic Environment	1.26	0.171	7.376	***	par_1
HE4	←	Humanistic Environment	1.348	0.163	8.284	***	par_2
HE3	←	Humanistic Environment	1.325	0.16	8.266	***	par_3
HE2	←	Humanistic Environment	1.165	0.136	8.543	***	par_4
BS2	←	Bodily Sensation	0.934	0.108	8.637	***	par_5
BS3	←	Bodily Sensation	1.016	0.089	11.351	***	par_6
BS4	←	Bodily Sensation	1.139	0.098	11.628	***	par_7
BS1	←	Bodily Sensation	1				
HE1	←	Humanistic Environment	1				
HE6	←	Humanistic Environment	1.429	0.177	8.059	***	par_8
BP6	←	Building Performance	0.855	0.084	10.183	***	par_9
BP5	←	Building Performance	0.906	0.078	11.613	***	par_10
BP4	←	Building Performance	1.064	0.083	12.885	***	par_11
BP3	←	Building Performance	0.802	0.087	9.191	***	par_12
BP1	←	Building Performance	1				
BP2	←	Building Performance	0.972	0.075	13.036	***	par_13
BP7	←	Building Performance	0.94	0.084	11.241	***	par_14

*** *p* < 0.001.

**Table 6:** Verified results of the proposed hypotheses.

Hypothesis	Description	Yes/No
H1	“Building Performance” has a significant positive impact on “Bodily Sensation”	Y
H2	“Building Performance” has a significant positive impact on “Humanistic Environment”	N
H3	“Bodily Sensation” has a significant positive impact on “Humanistic Environment”	Y
H4	“Building Performance” has a significant positive impact on the development of a healthy dormitory	Y
H5	“Bodily Sensation” has a significant positive impact on the development of a healthy dormitory	Y
H6	“Humanistic Environment” has a significant positive impact on the development of a healthy dormitory	Y

## Supplementary Materials

The following are available online at https://www.mdpi.com/1660-4601/17/5/1565/s1, supplementary material: Formal questionnaire.

## References

[1] WHO Healthy China 2030 (from Vision to Action). https://www.who.int/healthpromotion/conferences/9gchp/healthy-china/en/.

[2] Li H.Y., Zhang X.L., Ng S.T., Skitmore M. (2018). Quantifying stakeholder influence in decision/evaluations relating to sustainable construction in China—A Delphi approach. J. Clean. Prod..

[3] Wu Z., Jiang M., Cai Y., Wang H., Li S. (2019). What Hinders the Development of Green Building? An Investigation of China. Int. J. Environ. Res. Public Health.

[4] Wu Z., Li H., Feng Y., Luo X., Chen Q. (2019). Developing a green building evaluation standard for interior decoration: A case study of China. Build. Environ..

[5] Tam V.W.Y., Karimipour H., Le K.N., Wang J. (2018). Green neighbourhood: Review on the international assessment systems. Renew. Sustain. Energy Rev..

[6] ASC (2017). Assessment Standard for Healthy Building.

[7] Mao P., Qi J., Tan Y.T., Li J. (2017). An examination of factors affecting healthy building: An empirical study in east China. J. Clean. Prod..

[8] IWBI Buildings and Communities that Help People Thrive. https://www.wellcertified.com/certification/v2/.

[9] Heinzerling D., Schiavon S., Webster T., Arens E. (2013). Indoor environmental quality assessment models: A literature review and a proposed weighting and classification scheme. Build Environ..

[10] Piasecki M. (2019). Practical Implementation of the Indoor Environmental Quality Model for the Assessment of Nearly Zero Energy Single-Family Building. Buildings.

[11] Piasecki M., Kostyrko K.B. (2019). Combined Model for IAQ Assessment: Part 1—Morphology of the Model and Selection of Substantial Air Quality Impact Sub-Models. Appl. Sci..

[12] Alfonsin N., McLeod V., Loder A., DiPietro L. (2018). Active Design Strategies and the Evolution of the WELL Building Standard (TM). J. Phys. Act. Health.

[13] McLeod V. (2018). The WELL Building Standard (TM) (WELL): The intersection of physical activity and the built environment. J. Phys. Act. Health.

[14] Fan B. (2015). Analysis of University Student Apartment Design. Ph.D. Thesis.

[15] Lv L.G., Wang Y.L., Zhang P., Jin Y.W. (2010). Investigation and Assessment on Thermal Environment of University Dormitory in Luoyang. Build. Energy Effic..

[16] Zhu S., Tan H., Chen S., Zhuang Z. (2014). Situation and analysis of green campus culture construction in China. Build. Energy Effic..

[17] Petidis I., Aryblia M., Daras T., Tsoutsos T. (2018). Energy saving and thermal comfort interventions based on occupants’ needs: A students’ residence building case. Energy Build..

[18] Lu M., Ge J., Chen S., Qu L., Chen W. (2016). Current situation analysis of policies related to promote green campus construction in China. Archit. Technol..

[19] Kilicaslan H. (2013). Design of Living Spaces in Dormitories. Procedia Soc. Behav. Sci..

[20] Ding Z., Hu T., Li M., Xu X., Zou P.X.W. (2019). Agent-based model for simulating building energy management in student residences. Energy Build..

[21] He Y., Li N., Peng J., Zhang W., Li Y. (2016). Field study on adaptive comfort in air conditioned dormitories of university with hot-humid climate in summer. Energy Build..

[22] Lei Z., Liu C., Wang L., Li N. (2017). Effect of natural ventilation on indoor air quality and thermal comfort in dormitory during winter. Build Environ..

[23] Wang F., Wang J., Han M., Jia C., Zhou Y. (2019). Heavy metal characteristics and health risk assessment of PM2.5 in students’ dormitories in a university in Nanjing, China. Build Environ..

[24] Frijters P., Islam A., Pakrashi D. (2019). Heterogeneity in peer effects in random dormitory assignment in a developing country. J. Econ. Behav. Organ..

[25] Pilechi P., Taherkhani P. (2011). Social Sustainability in Student Dormitories. Procedia Eng..

[26] Maslow A., Lewis K.J.J.S.I. (1987). Maslow’s hierarchy of needs. Salenger Inc..

[27] McLeod S. Maslow’s Hierarchy of Needs. https://www.simplypsychology.org/maslow.html.

[28] Wahba M.A., Bridwell L.G. (1973). Maslow reconsidered: A review of research on the need hierarchy theory. Organ. Behav. Hum. Perform..

[29] Pinho P.G., Pinto M., Almeida R.M.S.F., Lopes S.M., Lemos L.T. (2016). Aspects concerning the acoustical performance of school buildings in Portugal. Appl. Acoust..

[30] ISO ISO1996: Acoustics—Description, Measurement and Assessment of Environmental Noise—Part 1: Basic Quantities and Assessment Procedures. https://www.iso.org/standard/59765.html.

[31] Alfano R.D., Dell’Isola M., Ficco G., Palella B., Riccio G. (2016). Experimental Air-Tightness Analysis in Mediterranean Buildings after Windows Retrofit. Sustainability.

[32] ISO ISO 9972: Thermal Performance of Buildings—Determination of Air Permeability of Buildings—Fan Pressurization Method. https://www.iso.org/standard/55718.html.

[33] Charde M., Gupta R. (2013). Effect of energy efficient building elements on summer cooling of buildings. Energy Build..

[34] Boubekri M., Lee J. (2017). A comparison of four daylighting metrics in assessing the daylighting performance of three shading systems. J. Green Build..

[35] Asfour O.S. (2017). Effect of building plan form on human thermal comfort in naturally ventilated open-plan enclosures located in hot climates. J. Green Build..

[36] Bellia L., Fragliasso F., Stefanizzi E. (2017). Daylit offices: A comparison between measured parameters assessing light quality and users’ opinions. Build. Environ..

[37] ISO ISO 10916: Calculation of the Impact of Daylight Utilization on the Net and Final Energy Demand for Lighting. https://www.iso.org/standard/46394.html.

[38] Yoon S.-H., Kim S.-Y., Park G.-H., Kim Y.-K., Cho C.-H., Park B.-H. (2018). Multiple power-based building energy management system for efficient management of building energy. Sustain. Cities Soc..

[39] Wei Y., Li Y., Liu X., Wu M. (2019). Sustainable development and green gross domestic product assessments in megacities based on the emergy analysis method—A case study of Wuhan. Sustain. Dev..

[40] ISO ISO 23045: Building Environment Design—Guidelines to Assess Energy Efficiency of New Buildings. https://www.iso.org/standard/45694.html.

[41] Arpke A., Strong K. (2006). A comparison of life cycle cost analyses for a typical college dormitory using subsidized versus full-cost pricing of water. Ecol. Econ..

[42] Huang L., Liu Y., Krigsvoll G., Johansen F. (2018). Life cycle assessment and life cycle cost of university dormitories in the southeast China: Case study of the university town of Fuzhou. J. Clean. Prod..

[43] Aguilera J.J., Kazanci O.B., Toftum J. (2019). Thermal adaptation in occupant-driven HVAC control. J. Build. Eng..

[44] Alfano R.D., Dell’Isola M., Ficco G., Palella B.I., Riccio G., Frattolillo A. (2019). Thermal comfort in Supermarket’s refrigerated areas: An integrated survey in central Italy. Build. Environ..

[45] ISO ISO 10551: Ergonomics of the Physical Environment—Subjective Judgement Scales for Assessing Physical Environments. https://www.iso.org/standard/67186.html.

[46] Kellert S.R., Fath B. (2008). Biophilia. Encyclopedia of Ecology.

[47] Xue F., Lau S.S., Gou Z., Song Y., Jiang B. (2019). Incorporating biophilia into green building rating tools for promoting health and wellbeing. Environ. Impact Assess. Rev..

[48] Andersen R.K., Fabi V., Corgnati S.P. (2016). Predicted and actual indoor environmental quality: Verification of occupants’ behaviour models in residential buildings. Energy Build..

[49] Wei Y., Gu J., Wang H., Yao T., Wu Z. (2018). Uncovering the culprits of air pollution: Evidence from China’s economic sectors and regional heterogeneities. J. Clean. Prod..

[50] Radwan A., Issa M.H. (2017). An evaluation of indoor environmental quality and occupant well-being in Manitoba school buildings. J. Green Build..

[51] Krešić G., Šimundić B., Mandić M.L., Kenđel G., Pavičić Žeželj S. (2008). Daily menus can result in suboptimal nutrient intakes, especially calcium, of adolescents living in dormitories. Nutr. Res..

[52] Shook N.J., Clay R. (2012). Interracial roommate relationships: A mechanism for promoting sense of belonging at university and academic performance. J. Exp. Soc. Psychol..

[53] Baihua X. (2008). Student Moral Education and Conduct Instruction in Student Dormitory Management in Institution of Higher Learning. Bull. Sci. Technol..

[54] Zhao X., Zhu N. (2019). An Investigation into the Interpersonal Relationship in College Students’ Dormitories. Chin. J. Spec. Educ..

[55] Jian H. (2012). A Probe into Promoting the Efficiency of the Administration of Freshmen’s Dormitory. Energy Procedia.

[56] Pedaste M., Pedaste K., Lukk K., Villems P., Allas R. (2014). A Model of Innovation Schools: Estonian Case-study. Procedia Soc. Behav. Sci..

[57] Cuce E., Sher F., Sadiq H., Cuce P.M., Guclu T., Besir A.B. (2019). Sustainable ventilation strategies in buildings: CFD research. Sustain. Energy Technol. Assess..

[58] Kisilewicz T., Fedorczak-Cisak M., Barkanyi T. (2019). Active thermal insulation as an element limiting heat loss through external walls. Energy Build..

[59] IBM IBM SPSS Software. https://www.ibm.com/analytics/spss-statistics-software.

[60] IBM Amos Structural Equation Modeling. https://www.ibm.com/us-en/marketplace/structural-equation-modeling-sem.

[61] Eisinga R., Grotenhuis M.T., Pelzer B. (2013). The reliability of a two-item scale: Pearson, Cronbach, or Spearman-Brown?. Int. J. Public Health.

[62] Wu Z., Yu A.T.W., Wang H., Wei Y., Huo X. (2019). Driving factors for construction waste minimization: Empirical studies in Hong Kong and Shenzhen. J. Green Build..

[63] Li H. (2000). Methodology of Management Research.

[64] Chang T.Y., Deng X.P., Hwang B.G., Zhao X.J. (2019). Improving Quantitative Assessment of Political Risk in International Construction Projects: The Case of Chinese Construction Companies. J. Constr. Eng. Manag..

[65] Wu Z., Yu A.T.W., Shen L. (2017). Investigating the determinants of contractor’s construction and demolition waste management behavior in Mainland China. Waste Manag..

[66] Hsu I.Y., Su T.-S., Kao C.-S., Shu Y.-L., Lin P.-R., Tseng J.-M. (2012). Analysis of business safety performance by structural equation models. Saf. Sci..

[67] Dell’Olio L., Ibeas A., Oña J.D., Oña R.D. (2018). Chapter 8—Structural Equation Models. Public Transp. Qual. Serv..

[68] Hoisington A.J., Stearns-Yoder K.A., Schuldt S.J., Beemer C.J., Maestre J.P., Kinney K.A., Postolache T.T., Lowry C.A., Brenner L.A. (2019). Ten questions concerning the built environment and mental health. Build Environ..

[69] Evans G.W. (2003). The built environment and mental health. J. Urban Health.

[70] Watkins D.C., Hunt J.B., Eisenberg D. (2012). Increased demand for mental health services on college campuses: Perspectives from administrators. Qual. Soc. Work.

