# Supplementary material for: Investigating the Crucial Aspects of Developing a Healthy Dormitory based on Maslow’s Hierarchy of Needs—A Case Study of Shenzhen
Source: Int J Environ Res Public Health. 2020 Feb 28;17(5):1565. doi: 10.3390/ijerph17051565 (PMC7094210; doi:10.3390/ijerph17051565)
Supplement: Supplementary file 1 [file ijerph-17-01565-s001.pdf]

International Journal of
Environmental Research and Public Health

MDPI

Article

Investigating the Crucial Aspects of Developing a Healthy Dormitory based on Maslow's Hierarchy of Needs—A Case Study of Shenzhen

Zezhou Wu [1,2], Lei Liu [2], Shenghan Li [1,2,*], and Hao Wang [3]

1. Sino-Australia Joint Research Centre in BIM and Smart Construction, Shenzhen University, S**henzhen 518060, China; wuzezhou@szu.edu.cn**
2. Department of Construction Management and Real Estate, College of Civil and Transportation Engineering, Shenzhen University, Shenzhen 518060, China; leiliu940610@163.com
3. School of Management Science and Engineering, Central University of Finance and Economics, Beijing, 100081, China; holy.wong@connect.polyu.hk

* Correspondence: shenghan@szu.edu.cn; Tel.: +86-755-2653-5406

Received: 11 February 2020; Accepted: 21 February 2020; Published: date

Supplementary material: Formal questionnaire

Part 1: Your basic information

1. What is your sex?		□Male	□Female	
2. What is your category?	□Undergraduate	□Master	□Doctor	□Staff
3. Which building do you live in?	□Liyuan	□Xiyuan	□Qiaoyuan	□Nnanyuan

Part 2: Considerations of healthy dormitory

□ Strongly disagree "1" □ Disagree "2" □ Neutral "3" □ Agree "4" □ Strongly agree "5"

**Building Performance**					
1. Suitable materials should be selected to enhance the sound insulation of walls and floors.	1	2	3	4	5
2. The gas tightness of the outer doors and windows should be improved to reduce the heat loss caused by the air penetration.	1	2	3	4	5
3. Without affecting the lighting, excessive heat radiation should be prevented from entering the room.	1	2	3	4	5
4. Appropriate active equipment and passive designs should be selected to collect and store light energy.	1	2	3	4	5
5. The spatial layout should be redesigned to promote air flow by increasing the difference between indoor and outdoor wind pressure and temperature.	1	2	3	4	5
6. Life-cycle costs should be calculated to measure economic benefits and determine its sustainable potential.	1	2	3	4	5
7. Energy consumption should be monitored in real time, intelligently controlled and managed.	1	2	3	4	5
**Bodily Sensation**					
1. Indoor thermal conditions should be improved such as temperature, air quality and humidity.	1	2	3	4	5
2. New greening forms and bio-diversity should be increased to alleviate visual fatigue in students.	1	2	3	4	5
3. It is the air quality within and around buildings and structures. Poor indoor air quality has been linked to sick building syndrome, reduced productivity and impaired learning in schools.	1	2	3	4	5
4. Healthy food stores/markets should be provided, and healthy eating habits also should be encouraged.	1	2	3	4	5
**Humanistic Environment**					

1. Abundant service facilities make students' outdoor activities more convenient.	1	2	3	4	5
2. Group activities with unfamiliar students can promote a harmonious atmosphere in the dormitory area.	1	2	3	4	5
3. Actively promoting courses related to physical and mental health and energy saving knowledge.	1	2	3	4	5
4. Students can choose their own dormitory according to their personal characteristics and living habits.	1	2	3	4	5
5. Evaluation policies and reward policies should be actively promoted, such as full-time teachers' visits and psychological counseling.	1	2	3	4	5
6. Innovative thinking, technology and methods are important driving force for the development of dormitory environment.	1	2	3	4	5
